# Traces of Meaning Itself: Encoding Distributional Word Vectors in Brain Activity

**DOI:** 10.1162/nol_a_00003

**Published:** 2020-03-01

**Authors:** Jona Sassenhagen, Christian J. Fiebach

**Affiliations:** Department of Psychology, Goethe University Frankfurt, Germany; Department of Psychology, Goethe University Frankfurt, Germany; Brain Imaging Center, Goethe University Frankfurt, Germany

**Keywords:** semantics, EEG, Word2vec, N400, encoding/decoding, MVPA

## Abstract

How is semantic information stored in the human mind and brain? Some philosophers and cognitive scientists argue for vectorial representations of concepts, where the meaning of a word is represented as its position in a high-dimensional neural state space. At the intersection of natural language processing and artificial intelligence, a class of very successful distributional word vector models has developed that can account for classic EEG findings of language, that is, the ease versus difficulty of integrating a word with its sentence context. However, models of semantics have to account not only for context-based word processing, but should also describe how word meaning is represented. Here, we investigate whether distributional vector representations of word meaning can model brain activity induced by words presented without context. Using EEG activity (event-related brain potentials) collected while participants in two experiments (English and German) read isolated words, we encoded and decoded word vectors taken from the family of prediction-based Word2vec algorithms. We found that, first, the position of a word in vector space allows the prediction of the pattern of corresponding neural activity over time, in particular during a time window of 300 to 500 ms after word onset. Second, distributional models perform better than a human-created taxonomic baseline model (WordNet), and this holds for several distinct vector-based models. Third, multiple latent semantic dimensions of word meaning can be decoded from brain activity. Combined, these results suggest that empiricist, prediction-based vectorial representations of meaning are a viable candidate for the representational architecture of human semantic knowledge.

## INTRODUCTION

How is the meaning of words represented in the human mind? A range of neurocognitive models discuss the neural underpinnings of semantic representation (see Borghesani & Piazza, 2017 for a recent review), focusing primarily on studying the neural localization of semantic processing (e.g., Lambon Ralph & Patterson, 2008; Patterson, Nestor, & Rogers, 2007) or its temporal organization (Kutas & Federmeier, 2000; Lau, Phillips, & Poeppel, 2008). Yet we have so far gained comparatively little insight into how semantic meaning is represented in our mind. From a psychological or linguistic perspective, the methods of cognitive neuroscience can, however, also be used fruitfully for investigating the “nature” of semantic representations. For example, functional mapping of brain activation (using methods such as functional MRI [fMRI] or event-related potentials [ERPs] of the human electroencephalogram) has established that semantic features like the concreteness versus abstractness of a word reflect in distinguishable neural signatures elicited during lexical processing (e.g., Fiebach & Friederici, 2004; Krause, Aström, Karrasch, Laine, & Sillanmäki, 1999). Similarly, in some contexts, action-associated words have been shown to co-activate brain regions associated with the corresponding motor representations (Hauk, Johnsrude, & Pulvermüller, 2004), whereas concrete and imageable words co-activate sensory brain regions (e.g., Aziz-Zadeh & Damasio, 2008; Aziz-Zadeh et al., 2008). And at the level of sentence semantics, it was, for example, demonstrated that abstract semantic and experiential world knowledge are processed similarly (e.g., Hagoort, Hald, Bastiaansen, & Petersson, 2004).

However, rather than providing direct insights into the nature of semantic representations, these and many other neurolinguistics studies are restricted to testing indirect implications of particular aspects of theories of semantic meaning, often in specific processing contexts (e.g., during the comprehension of incongruent sentences). But theories of semantics have to account for both the mental representation of semantic concepts and how they are processed. Our understanding of the nature of semantic representations is thus not sufficiently constrained by the mere fact that, for example, concrete and abstract words are not processed “identically” in the human brain. Put differently, empirical work has managed to demonstrate that brains at the very least encode concreteness, but this insight does not further our understanding of how semantic information is represented. This argument extends beyond concreteness effects to all domains of semantic knowledge. Only recently have theoretical and methodological developments as well as the availability of novel data sets made it possible to address more directly outstanding questions regarding fundamental aspect of semantics, including the nature of semantic representations.

An empirical investigation of what aspects of semantic meaning are represented and how they are represented, requires that the to-be-tested models are quantitatively explicit. This is, however, typically not the case for psychological or neurolinguistic models of semantics such as classical symbolic theories of meaning (Fodor, 2004), prototype theories of meaning (Rosch, 1999), embodied cognition theories of language (Pulvermüller, 2013), or neuropsychological models like the hubs and spokes model of semantics (Lambon Ralph & Patterson, 2008). This contrasts with the field of computerized natural language processing (e.g., Jurafsky & Martin, 2014), which has in recent years seen a very dynamic development of strongly empiricist feature–based machine learning models of semantics. Models like the so-called “Word2vec” family (Mikolov, Chen, Corrado, & Dean, 2013; Pennington, Socher, & Manning, 2014) have been applied successfully to most domains of language processing, like sentiment analysis (Felbo, Mislove, Søgaard, Rahwan, & Lehmann, 2017), machine translation (Mikolov, Le, & Sutskever, 2013), and document retrieval (Ju, Zhou, Li, & Liu, 2015). Here, we propose that machine learning techniques (Hastie, Tibshirani, & Friedman, 2009) can be leveraged to “directly” test the psychological plausibility of such quantitative theories of semantic representation by assessing their fit with neuroimaging results, and that this will advance our understanding of the nature of semantic representations.

### Prediction-based Distributional Models and Semantic Knowledge

**Figure 1.**
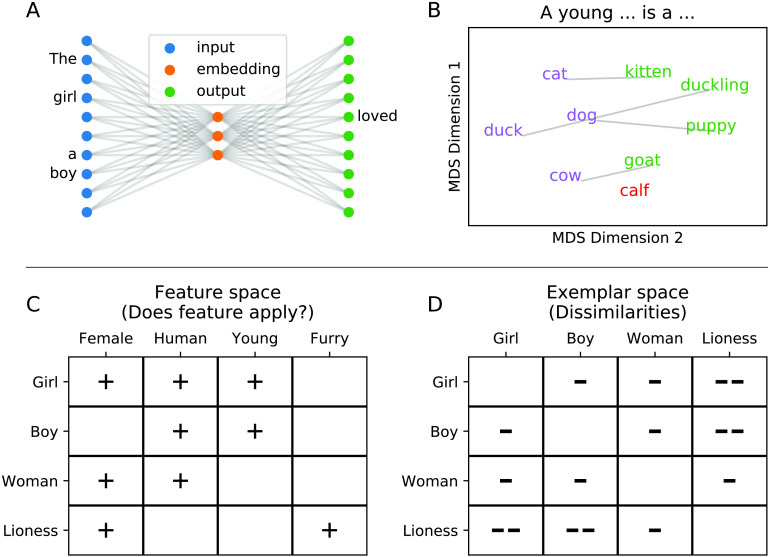
Vectorial models of semantic word knowledge. *Top*: Architecture and example content of distributional models. A. Basic architecture of a so-called Continuous Bag of Words prediction model for word embeddings. An input layer (*blue*) receives a context, and aims to learn weights on the internal layer (*orange*), which in turn allows the prediction of the omitted target word (*green*); read from right to left, this corresponds to a Skip-Gram model. B. Vectorial models can learn semantics-like relationships. For purposes of illustration, we looked up vectors V in a pretrained vector model (Mikolov, Grave, Bojanowski, Puhrsch, & Joulin, 2017), for four animals (*cat*, *dog*, *cow*, and *duck*). We then added to them the vector for the word “young,” and retrieved the word closest to the resulting vector, resulting in “kitten,” “puppy,” “goat,” and “duckling,” respectively. Only for the word cow, this calculation of V_cow_ + V_young_ did not result in a young instance of the species cow (i.e., a calf). The model, accordingly, is capable of roughly representing semantic relationships such as “a young W_1_ is a W_2_.” For visualization purposes, these eight words plus the missing response “calf” were embedded into two dimensions with Multidimensional Scaling as implemented in scikit-learn (Pedregosa et al., 2011). C, D. Demonstration of feature (C) and exemplar-based (D) representations of concepts; “+” indicates the presence of a feature (*left*) and the number of “−”s represents the dissimilarity between two concepts (*right*).

How do distributional models of the Word2vec family function? Mikolov et al. (2013) introduced a prediction-based word-embedding model implemented as a simple single-layer neural network that learns to predict a withheld target word given a 10-word context (i.e., the 5-word strings preceding and following the target in the training corpus, independent of any grammatical constraints). This corresponds to the Continuous Bag of Words version of the Word2vec model. The inverse—that is, predicting a context from a target word—is the Skip-Gram model. As Mikolov et al. (2013) describe, both perform approximately equivalently. The input and output spaces of such networks are large: If trained on sources such as the English Wikipedia, they contain more than 100,000 entries (i.e., unique words). Of interest, their internal structure is, however, very simple—comprising only a few tens or hundreds of neurons (often 300; see, e.g., Figure 1A for a schematic illustration of the model architecture). We hypothesized that this “many-to-few” compression may resemble the manner in which the human brain learns and represents semantic knowledge. Accordingly, we test the relationship between brain signals elicited during word processing and the vectorial representations of these words resulting from empiricist learning models like Word2vec.

Artificial learning systems, under which distributional models of semantics can be subsumed, resemble human learners in that in the interest of generating abstract, generalizable inferences, they benefit from constraints on their capacity (Elman, 1991). This is also a crucial factor for Word2vec family models, which perform better when the internal network size is smaller (Landauer & Dumais, 1997; Mikolov et al., 2013). Under such situations of limited internal “representational capacity,” the prediction-based training regimen forces the internal layer, which is unable to simply “memorize” all mappings, to discover latent semantic dimensions (Baroni, Dinu, & Kruszewski, 2014; Landauer & Dumais, 1997). This “compression” is essential to word-embedding models. Consider, as an example, the relationship between the (African) lion and the (Asiatic) tiger, that rarely appear at physically identical places in their natural environments. An unconstrained representation (i.e., a manually annotated expert system like the WordNet of Miller [1995]) would represent isolated facts like “lions are mammals” (or +MAMMAL), “tigers are mammals,” “lions are quadrupeds,” and so on (see Figure 1C for schematic). In contrast, a prediction-based and capacity-constrained distributional model would have to identify what is shared between lions and tigers (e.g., that they are large cats and occur in similar text contexts) and code this information in an abbreviated, synergistic fashion. This strategy of reducing a data set, such as the co-occurrence statistics of words, to a limited number of latent, generalizable factors underlying its axes of variance, is particularly well suited for uncovering such implicit, underlying similarities. These “distributed” and “distributional” models are indeed capable of learning word meaning from distributional information only (see also Landauer & Dumais, 1997). As Figure 1B schematically demonstrates, they are even capable of tasks such as compositionality (see also Mikolov et al., 2013), which is generally considered a key aspect of any theory of semantics (Fodor & LePore, 1999).

### Vectors in the Brain

After training a distributional network model, the weights between each word and the, for example, 300 neurons in the hidden layer are understood as an “embedding” of the word, so that each word is described as a vector of 300 values (neural weights) corresponding to the axes of the vector space. This is equivalent to a position in a high-dimensional state space (Churchland, 1993). The vectors are dense and real-valued. That is, unlike typical hand-designed feature space models in which only very few words carry a feature, such as +HUMAN or +QUADRUPED, in a typical word embedding every word ranks somewhere on every axis. These axes are generally not directly interpretable, and do not typically correspond to natural-language concepts (like animacy). Instead, semantic knowledge is coded in a distributed manner, so that many axes will each be partially correlated with any given semantic dimension (Landauer & Dumais, 1997). One way for making visible the semantic knowledge in these models is to project them into exemplar space, for example, by computing the correlation between all items. The resulting item-by-item representation shows how similar each concept is to all other concepts (see Figure 1D), which corresponds to a “kernel”-based representation of the semantic space (see Discussion).

Embedded distributional models are highly effective in accounting for human processing of words in contexts. More specifically, they can predict priming effects (Günther, Dudschig, & Kaup, 2016a, 2016b), human behavioral performance in multiple other psycholinguistic tasks (Mandera, Keuleers, & Brysbaert, 2017), semantic association ratings (Rubinstein, Levi, Schwartz, & Rappoport, 2015), similarity ratings (Mandera et al., 2017), and ERP measures of the fit of a word with its context (Broderick, Anderson, Di Liberto, Crosse, & Lalor, 2018; Ettinger, Feldman, Resnik, & Phillips, 2016). But that is almost by design, as distributional models are trained by finding patterns in item-context pairs (Levy & Goldberg, 2014), and the observation above, that is, that context effects provide only indirect insight into the nature of semantic representations, also applies to empirical tests of distributional models of word semantics as long as they are based on item-context associations. In the present study, we go one step further and explore whether distributional models can directly account also for the neurocognitive “representation” of word meaning in the human brain. To this end, we postulate that understanding the meaning of a word is equivalent to transitioning the brain into a (more or less short-lived) unique state that systematically depends on the meaning of the perceived word. If this hypothesis is true, it should be possible to do the following. First, predict not only behavior (see above), but also brain activity based on word vectors; second, predict not only item-context or item-item effects, but also neural correlates of context-free word processing; and third, conversely, partially recover a word’s position in vector space from brain activity elicited by the respective word.

### Relation to Prior Work

A number of recent studies have used vectorial models to identify neural correlates of sets of semantic categories or features (e.g., Gauthier & Ivanova, 2018; Kemmerer, Castillo, Talavage, Patterson, & Wiley, 2008; Mitchell et al., 2008; Pereira et al., 2018; Sudre et al., 2012; Wehbe et al., 2014; Xu, Murphy, & Fyshe, 2016). Recently, Huth, de Heer, Griffiths, Theunissen, and Gallant (2016) have shown, for example, that it is possible to predict with high accuracy brain activity elicited during listening to narratives. This work has revealed a distributed “tiling” of semantic features across the cortex, and a particular sensitivity to categories such as the social relevance of words.

In contrast to this seminal work, here we aim at using the framework of distributional models to examine the neural representation of word meanings “themselves”—that is, the neural signatures of words bereft of context, and the hypothesized systematic relationship to their position in semantic vector space as quantified with distributional models learned in an empiricist fashion from statistical patterns of co-occurrence alone. Specifically, we tested whether word-associated brain activity can be predicted—“encoded”—from Word2vec-style distributional vector representations of these words. We found that different vector spaces could be encoded successfully in brain activity. Second, we explored as a proof-of-principle what information about word vectors is contained in the electrophysiological activity of the brain elicited during word processing, by testing whether the word’s position in (for the sake of interpretability, dimensionality-reduced) vector space can be “decoded” from brain activity. Then, we established conceptual labels for the aspects of word vectors decodable from brain activity. This latter stage was conducted to ensure that brain activity was correlated with the “semantic” information in distributional models, not (or at least not exclusively) with other information about words that these models might contain (e.g., about word frequencies or similar lexical properties of words). We indeed observed that for components corresponding to semantic dimensions, vector-space factor scores of words could be read out from brain activity. Taken together, the work presented here is an initial demonstration that it is plausible that brain states elicited by words are approximately isomorphic to the position of these words in a distributional model of word meaning.

## METHODS

### Data Sets and Preprocessing

We herein report results from two data sets, acquired in two different languages and using two different tasks: The first dataset contains ERP data for 960 visually presented English nouns (*n* = 75, 28 EEG channels; for details see Dufau, Grainger, Midgley, & Holcomb, 2015). Words were shown on a screen for 400 ms, followed by a 600-ms blank screen, and were mixed with 140 nonword probes requiring a manual response (discarded from analysis). The second data set was acquired in our own lab and involves EEG signals elicited by 150 visually presented German nouns (presented for 1000 ms following a 500-ms fixation cross; *n* = 35; for details see https://www.biorxiv.org/content/10.1101/393066v1). In this experiment, for each word, participants were instructed to press a button if and only if it was synonymous with the previous word (the 10 synonymous probes requiring a button press were ignored in this analysis). EEG was collected from 64 ActiCap active electrodes via a Brainamp amplifier (Brainproducts; Gilching, Germany).

Data analysis was conducted in MNE-Python (Gramfort et al., 2013). The English data set was obtained from the original authors in a preprocessed form, in the form of per-word average ERPs across subjects (−100- to 920-ms post word onset; see Dufau et al., 2015). For the German data set, eye movement artifacts were removed via independent component analysis (Jung et al., 2000), and a .1 to 30 Hz bandpass filter was applied. Both data sets were downsampled to 200 Hz. Then, analogous to the English data set, average ERPs across subjects were calculated individually for each word for the German data. Compared to single-subject analysis and averaging, this approach leads to higher signal-to-noise ratios (SNRs), but does not change the overall pattern of results.

We note informally that we have applied the same analysis to a series of other data sets; the results we show here can be replicated on a number of other sufficiently large data sets of ERPs elicited during single-word presentation.

### Word Embeddings

For all following analyses, for the vector semantics, we relied on FastText, that is, a further development of the above-described Word2vec algorithm (Bojanowski, Grave, Joulin, & Mikolov, 2016). We used the implementation from the natural language processing package GenSim (Řehŭřek & Sojka, 2011) and for both German and English, the publicly available, pretrained, 300-dimensional embeddings provided by Mikolov et al. (2017). Note that FastText’s primary improvement upon Word2vec is that by accounting for units below the word level, it naturally handles inflected forms. Due to this, it can better account for morphology-rich languages like German. Further machine learning (decoding/encoding) was conducted via sklearn (Pedregosa et al., 2011) and visualization done with Seaborn (Waskom et al., 2018), both using the Python programming language.

### Encoding Semantic Vectors in Brain Activity

We attempted to model the dependency of brain states on the position of the respective word in a semantic state space via an encoding model (King et al., in press). Specifically, we predicted brain activity by assuming that the neural state after having processed one word resembles a sum of neural states after having processed other words, weighted by the similarity of these words to the target word (if viewed as a “dual” problem; see below), or as a linear weighted sum of the features (vector-space axes). To provide a baseline model, we also conducted the same analysis based on a non-distributional model of word meaning. For this, we chose WordNet (Miller, 1995), a well-established database of lexical/semantic relations that contains a manually constructed taxonomy of words.

For both languages, within a 10-fold cross-validation loop, a Ridge regression (i.e., a linear regression with *L*_2_ norm regularization) was trained to predict the entire pattern of brain activity elicited by a word, across all measurement channels, using the respective word vectors as predictors. On each fold, the regression model used 90% of the data set as training trials to learn one coefficient for each of the vector-space model’s 300 dimensions, for each combination of time point and sensor. Subsequently, EEG activity (i.e., the pattern of signal amplitudes across sensors) was predicted for words not seen during training (i.e., the 10% trials held out in the respective fold), based on the dot product of the regression coefficients and the vector-embedding of the respective word. To account for the high temporal resolution of EEG, in each fold, independent regression models were fitted for each time point, and predictions on the test trials were also made per time point. The quality of these predictions were scored using the signed, squared correlation between predicted and observed EEG activity (i.e., ERPs), which provided an estimate of the systematic isomorphism between word vectors and electrophysiological brain activity.

**Figure 2.**
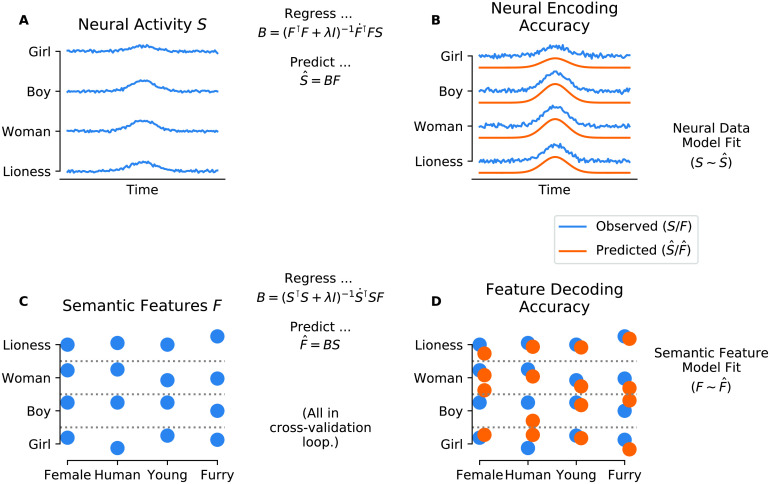
Decoding and encoding procedure, didactic example for simulated values. *Top*: Encoding procedure. A. Brain activity source data. For each word, the y-axis represents neural activity (i.e., microvolts in the case of EEG; ERP measurements). B. Comparison of predicted (*orange*) to the observed (*blue*) data. *Center*: A predictive model B is fit by estimating the coefficients minimizing a least-squares equation, to predict the ERP pattern (*S*) from the words’ semantic vector embedding *F. Bottom*: Decoding procedure. C. Interpretable semantic feature rankings of word vectors. Here, the y-axis represents the ranking on each of four exemplary semantic dimensions. D. Actual (*blue*) are compared to predicted (*orange*) semantic features, for each word. *Center*: In the decoding analysis, a predictive model B is fit by estimating the coefficients minimizing a least-squares equation, to predict semantic features F from ERP signal *S*.

These correlation coefficients were concatenated, resulting in one time series (from −100 to 920 ms time-locked to stimulus onset) per fold, corresponding to how well brain activity at each time point in the trial can be explained as a linear weighting of the word vectors for the respective word. These time courses were then averaged across folds, and 95% bootstrapped confidence intervals across folds were calculated for each time point. For a visualization of this procedure, see Figure 2. For statistical evaluation, first, the resulting prediction accuracy values were averaged in the N400 time window (300–500 ms; Lau et al., 2008), and a Wilcoxon signed-rank test against chance was conducted across folds. To control for potential biases, this was repeated for the average of the entire data epoch.

As noted, to compare the distributional word embedding model against a reasonable baseline model, we repeated the same analysis with similarities based on the WordNet taxonomy (Miller, 1995) for the English data set and, for the German data set, using a German equivalent (GermaNet; Hamp & Feldweg, 1997). WordNet’s taxonomy corresponds to hyponymy/hyperonymy relations—that is, “is-a” relationships like “a robin is a bird.” To predict EEG/ERP activity from this taxonomic model, we calculated “path similarities” for each pair of words, that is, the distance to the next shared hyperonym or hyponym. This led to a 960 × 960 matrix (English) and a 150 × 150 matrix (German), respectively, of semantic word/word distances, which were then used to predict EEG/ERP patterns across time points and electrodes and for checking the fit with actual ERPs on unseen trials, analogous to the FastText-based encoding described earlier (i.e., 10-fold cross-validation).

We calculated the difference, within each language, between prediction accuracy scores for the distributional/FastText-based predictions minus the score for WordNet- or GermaNet-based predictions. This was calculated separately for each fold, and a Wilcoxon rank-sum test on the average of the differences within each fold was conducted to establish which model yielded better predictions of the ERP patterns: the explicit taxonomic (i.e., WordNet/GermaNet) model, or the distributional model. Because the feature space built from WordNet is initially larger than that for FastText vectors for English (960 items × 960 items vs. 960 items × 300 dimensions) and smaller for German (150 × 150 vs. 150 × 300), to not bias the procedure in any direction, we computed this contrast also on a transformation of the FastText vector predictor array (*XX* ^⊺^), which has the same item × item dimensionality as the WordNet-derived matrix of path similarities. This yielded the same results (for possible reasons, see below the discussion of the Representer theorem).

Next, to rule out that successful encoding is the result of lexical but not semantic features being reflected in brain activity, we estimated if some of the encoding benefit for the superior model might reflect well-established psycholinguistic features like the frequency of occurrence of a word in a language, rather than the hypothesized greater similarity to the underlying neurocognitive correlates of semantics. To explore this alternative, we repeated the described analyses, but included in an exemplary manner word frequency (log counts per million as given by Dufau et al., 2015, for the English data; SUBTLEX log frequency, Brysbaert et al., 2011, for German) and concreteness (imaginability ratings from Dufau et al., 2015, for English and from Vo et al., 2009, for German) to the predictor arrays, and investigated if this influenced any of the observed effects. By doing so, we tested if any contrast between FastText-based and WordNet-based encoding performances was diminished as a result of adding explicit word frequency and concreteness values as predictors. We did not directly compare the predictive power of lexical variables like word frequency or concreteness to vector representations because this would entail comparing a very large predictor matrix (e.g., 960 predictors in English) to a much smaller one (i.e., with two predictor variables), so that much of the resulting differences might reflect the capacity of the algorithm to handle large feature spaces. In contrast, adding these psycholinguistic predictors to the set of baseline and word vector predictors and evaluating performance differences at the level of alternative models, reflects a meaningful test of the value of the word vector model.

Finally, to assess the specificity of our results for our specific choice of vector space, we repeated the encoding analysis for a set of other commonly used, pretrained distributional vector spaces, based on different algorithms (i.e., not from the Word2vec family), different training corpora, and different vector sizes. Specifically, we encoded the original Wikipedia-based Word2vec vectors released by Mikolov et al. (2013), the movie subtitle–based vectors by Mandera et al. (2017; Subtlex), and the full set of English vectors from Pennington et al. (2014) known as GloVe, based on a corpus incorporating texts obtained from the internet as well as Twitter messages. These embeddings encompassed multiple vector lengths (25–300).

### Decoding

In addition to encoding, a correspondence between the structure of brain activity and semantic spaces can be established by decoding—that is, by reading out properties of the stimulus based on brain activity. In our case, we tested if we could predict a word’s position in Word2vec space based on brain activity (see Figure 2, *bottom*).

Because the SNR of both the ERPs and the word vectors is low, predicting the absolute position of a word in high-dimensional vector space would lead to low fits and would accordingly be hard to interpret. We therefore first conducted dimensionality reduction on word vectors. For this, the vector space was transformed via Kernel Principal Component Analysis (PCA) with a cosine kernel (cosine similarity is the preferred distance measure for vector spaces, *cf*. Mikolov et al., 2013; see subsequent text for a discussion of kernels in vector semantics). We arbitrarily decided to investigate the first eight components (as all further components explain less variance and are more noisy and harder to interpret or read out of brain activity); that is, to investigate to which degree brain activity contains information about word semantics, we predicted each word’s loading on eight factors, resulting from Kernel PCA reduction of word vectors, from brain activity.

More specifically, we attempted to read out from brain activity elicited by words presented in isolation (same data as used for encoding analysis) the position of each word on each of these eight factors, using as predictors ERP time courses between 300 and 500 ms (reflecting the typical N400 time window; see preceding text). In a 10-fold cross-validation loop, Ridge regression was used to predict the position of each word in Kernel PCA–compressed vector space. Scoring was again accomplished via the signed, squared correlation (across items) between predicted and actually observed outcomes, resulting in 10 × 8 *r* ^2^ values—one per fold and factor—for each language (i.e., for each of the two datasets). For each factor, a 99.375% bootstrapped confidence interval was calculated, corresponding to a Bonferroni correction for the eight factors. In addition, a time-resolved multivariate decoding analysis (King & Dehaene, 2014) was conducted by repeating the same procedure as described earlier, but now separately for each sample time point of the full EEG data (with only electrode as feature). This resulted in 10 × 8 *r* ^2^ values for each time point, for each language. Sixty-eight percent bootstrapped confidence intervals were calculated (not corrected) for visualization only.

### Interpreting Word Vector Scores

As noted, the axes of distributional vector spaces do not a priori have interpretable labels, and are in fact difficult or impossible to label. Doubtlessly, the vector space as a whole contains a lot of semantic information about conceptual characteristics (like +ANIMACY). But this information is latent and must be actively inferred (which is, however, often not easily possible). In addition, semantic information is encoded in a distributed fashion; any semantic feature, such as animacy or concreteness, correlates with multiple axes of the vector space, but the specific axes themselves are not in a one to one relationship with any semantic trait. Thus, the decoding procedure described above could not—regardless of the success of decoding—indicate that information about word “meaning” is contained in brain activity. Any factor score that could be read out from brain activity could correspond to a number of nonsemantic aspects of word meaning also recoverable from vector spaces.

To rule out that a success in decoding vector-space position from brain activity may depend upon non-semantic aspects of word knowledge, we set out to label the eight factors used in the decoding analysis. Three trained linguists considered the seven words scoring most positively and most negatively on each of the eight components, separately for each language. Each linguist proposed one interpretation; then, each linguist ranked all three proposals for each axis. The proposal with the highest mean ranking was selected as a tentative label for the factor. Note that the specific labels are not important per se; the label interpretations are subjective in nature, and no transformation of the data is guaranteed to reproduce the “true” latent factor structure. Instead, this labeling was done simply to establish if brain activity allows decoding of “semantic” information at all—or if the correspondence between brain activity and distributional word vectors is due to non-semantic information contained in word vectors. That is, if brain activity allowed reading out the position of words on factors corresponding to conceptual distinctions, then this would speak for the decoding procedure succeeding because semantic information is encoded in brain activity.

### Visualizing the Spatial Structure of Encoding and Decoding

Finally, we set out to exploratorily visualize at which electrodes the signals on which encoder and decoder models operated were most clear. For this, we focused again on a time window of 300 to 500 ms after stimulus onset. We excluded the English data set because it was recorded with very sparse electrode coverage, and focused instead on the high-density German data. First, we aggregated the encoding performance over time within each electrode and visualized the result as an interpolated scalp map. Conceptually, this corresponds to a map of where the encoder procedure works best, that is, where whatever it is encoding is most directly represented. Next, we retrieved normalized regression coefficients for each Kernel PCA component from the decoding analysis, and similarly visualized them as scalp maps. Conceptually this corresponds to sites where higher activity predicts a higher (or lower, depending on the sign of the coefficient) component score, that is, where EEG/ERP activity is predictive of the respective component’s score per word.

## RESULTS

### Encoding Semantics in Brain Activity

**Figure 3.**
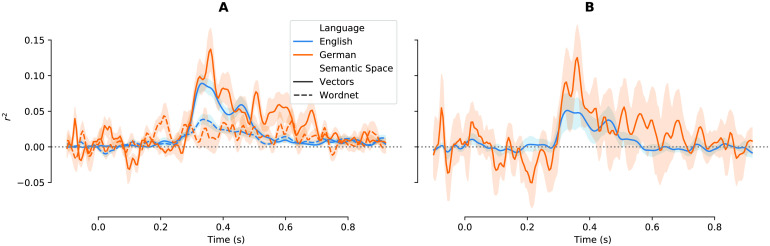
Encoding model fit for both languages. A. Predicting EEG activity based on word vectors. Goodness-of-fit across time, displayed as the squared and signed correlation between predicted and observed EEG signals, aggregating across sensors, participants, and folds, for word vector–embedding models (*solid lines*) and the WordNet (i.e., control) model (*dashed lines*) in English (*blue*) and the equivalent German models (*orange*). B. Fit improvement over WordNet. The difference between word vector models and taxonomic models demonstrates the significantly better prediction of EEG/ERP activity from word vector models. *Lines* represent the mean (squared, signed) correlation between the predicted and the actual ERP. *Shaded areas* reflect 68% bootstrapped confidence intervals over folds.

All four models (for English: FastText and WordNet path similarities; for German: German FastText and GermaNet path similarities) succeeded in predicting EEG activity, in particular around the N400 time window, which is known to reflect semantic memory access (see Figure 3, *left*; ∼250–550 ms). The vector-space models predicted activity well in our a priori predefined time window for statistical analyses (i.e., mean of 300–500 ms; English: *r* ^2^ = 0.05, SD = .025; German: *r* ^2^ = 0.07, SD = 0.045; both *p* < 0.005), but also when aggregating over the entire ERP epoch (both *p* < 0.005). It is important to note that vector-space models predicted brain activity significantly better than the taxonomic (WordNet) models (*p* < 0.01; compare *dashed* vs. *solid* lines in Figure 3). The right panel in Figure 3 displays the time-resolved improvement of semantic encoding based on vector space as compared to taxonomic models.

**Figure 4.**
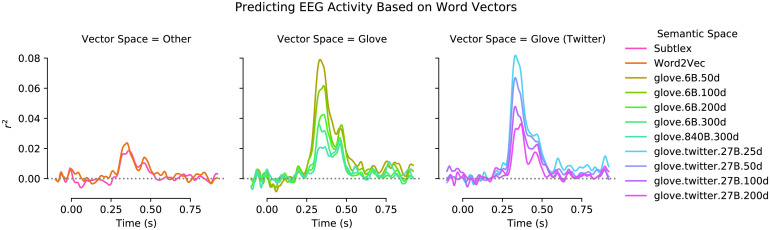
Encoding model fits for alternative (English) word vectors. All details as in Figure 3, but each plot shows the time series for encoding quality for multiple vector space models (see right legend, this figure). Word2vec and Subtlex vectors reflect an older algorithm and a different training corpus (movie subtitles), respectively. GloVe vectors (of various lengths) correspond to a related, but distinct algorithm (Pennington et al., 2014). GloVe vectors trained on Twitter data reflects yet another corpus. Note that although encoding accuracies vary across models, all models allow above-chance prediction of activity in the N400 time window.

Results for the alternative vector spaces are qualitatively similar (see Figure 4), indicating that distributional vectors in general allow prediction of brain activity. Of interest, shorter vectors appeared to allow superior encoding, potentially indicating overfitting for high-dimensional vectors.

### Decoding

Observing that encoding—that is, the prediction of brain activity during word reading based on the position of these words in vector space—is possible; we set out to more thoroughly test if this was due to the semantic content of word vectors. For this, we conducted a factor analysis (Kernel PCA) of word vectors (to support decoding and to render the results more interpretable), and then attempted to decode a word’s position in (factorized) vector space—that is, its loading on the components—based on brain activity. In addition, we subsequently aimed at inferring what these factors correspond to conceptually (see next section for details).

**Figure 5.**
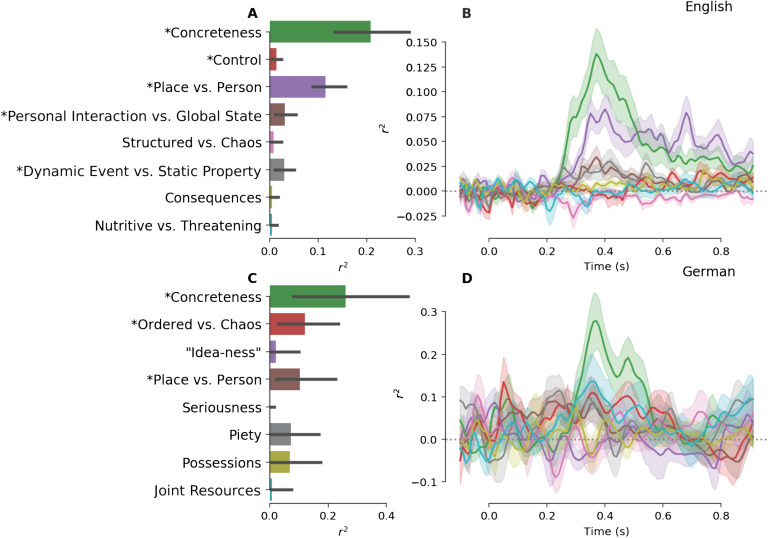
Decoding semantic dimensions from EEG data. A. Prediction accuracy (measured as the squared, signed correlations between predicted and actual values) for the prediction of eight vector-space factors from EEG activity between 300 and 500 ms, for English data. Labels on the y-axis reflect a subjective interpretation of the factor meanings (see Methods and Table 1). Error bars represent the 99.375% (95% corrected for multiple comparisons) bootstrapped confidence intervals; *asterisks* indicate decoding accuracies significantly different from chance. B. Time-resolved decoding scores for the same eight factors, when repeating the prediction at each sample time point of the EEG signal. Colors as on left plot. *Shaded outlines* reflect 68% bootstrapped confidence intervals over folds (uncorrected). C/D. Same, but for the German data set (note the different scaling of the y-axis in D as compared to B).

Decoding was used to predict the “loading” of each word on each of these eight factors, based on EEG activity. Results indicate that loadings on multiple factors could be read out from brain activity, with cross-validated (over 10-folds; see Methods) *r* ^2^ scores between predicted and observed factor scores > .1 (*p* < .05, Bonferroni corrected; Figure 5A, C). For example, the first component could be read out with significantly above-chance accuracy in both languages (see subsequent text for a more detailed discussion of which factors could be decoded. Note that confidence intervals were wider for German, which we attribute to the much smaller number of trials in the German experiment. Time-resolved decoding (Figure 5B, D) indicated that information about these factors was recoverable from brain activity beginning at ∼250 ms and peaking at ∼350 to 400 ms, that is, again in the time window of the N400, the best-established semantic ERP component.

### Kernel PCA Factor Structure

The first eight factors identified via Kernel PCA decomposition in the distributional models for each language are shown in Table 1. The semantic interpretation of these factors (provided by trained linguists; see Methods for details) is ad hoc, is not constrained by brain activity, and does not conclusively establish that these factors are intrinsic to the structure of the distributional models. Nevertheless, it is noteworthy that the first of these factors in both languages constituted one of the most fundamental dimensions of semantics discussed in the literature, that is, Concreteness, and that at least two other factors (Place vs. Person; Ordered/Structured vs. Chaos) appear in both languages. Even more importantly, these clearly semantic components could be read out from brain activity: We found significantly above-chance decoding scores for Concreteness (German and English Factor 1), Place vs. Person (English Factor 3, German Factor 4), and Ordered vs. Chaos (German Factor 2). In addition to these factors, decoding scores significantly (*p* < .05, Bonferroni corrected) exceeded chance for Factors 4 and 6 (English); see Figure 5A, C.

**Table 1.** Tentative Labels for Each Component Derived From the Dimensionality Reduction of Word Vector Space, Plus Most High/Low-Scoring Words

**Factors Names**	**Negative**	**Positive**
***English Data set***		
*Concreteness*	walnut, sprig, magnolia, pumpkin, lantern, pine, tumbler	effort, hope, validity, urgency, interest, reason, concern
*Control*	member, officer, attorney, director, senator, staff, student	breakage, swirl, deep, excess, wisp, rasp, sheer
*Place vs. Person*	location, area, floor, facility, site, section, design	poet, poesy, aunt, thug, heretic, uncle, buffoon
*Personal Interaction vs. Global State*	remember, guess, remark, welcome, wish, answer, quibble	disease, impurity, violence, growth, economy, culture, health
*Structured vs. Chaos*	theorem, piece, phrase, method, item, abstract, paper	disarray, trouble, ruin, despair, turmoil, east, midst
*Dynamic Event vs. Static Property*	scene, orgy, poem, disaster, crisis, skit, chaos	average, capacity, staff, weight, superior, extra, respect
*Consequences*	southern, modern, common, middle, east, original, cove	cash, bait, embargo, pressure, pump, subpoena, whip
*Nutritive vs. Threatening*	dinner, luncheon, evening, cuisine, gourmet, honey, beer	tentacle, enemy, position, dilemma, fracture, shaft, rift
***German Data set***		
*Concreteness*	hinweis, zweifel, fehler, zeitpunkt, verdacht, wunsch, beweis	mantel, huhn, ratte, knarre, pistole, decke, jacke
*Ordered vs. Chaos*	hass, mut, schwert, kampf, sand, fluch, hals	fahrer, agent, bank, wohnung, klinik, woche, firma
*Idea-ness*	idee, antwort, loesung, theorie, rede, guete, waffe	gast, bus, strand, flughafen, urlaub, onkel, ehemann
*Person vs. Place*	soldat, agent, waffe, onkel, ehemann, krieger, kumpel	strand, insel, osten, mitte, tuer, muell, naehe
*Seriousness*	jugend, freiheit, seele, stadt, rache, gnade, krieg	artikel, scheck, scherz, laut, knopf, hinweis, sack
*Piety*	stress, spass, lust, urlaub, miete, geduld, zucker	laut, himmel, osten, mitte, kreuz, schwert, legende
*Possessions*	guete, naehe, koma, muell, tuer, onkel, antwort	wert, papier, mantel, uniform, lauf, kleidung, platz
*Joint Resources*	muell, guete, naehe, talent, besitz, firma, stolz	nase, minute, termin, schlaf, fahrt, Montag, woche

Note that words are shown as they were fed to the word vector model (i.e., all lower cased). The seven most strongly negatively and most strongly positively scoring words are shown as example.

Again, we wish to emphasize here that the observed factor structure is in no way indicative of a true semantic feature space that we assume to be represented in the human brain, and choosing different analysis parameters could lead to very different factor structures. This analysis, thus, is merely a tool for verifying certain aspects of our methodology, intended to demonstrate that at least some of the predictive power of distributional models on ERP data stems from the genuinely semantic information they represent.

### Visualizing Decoding and Encoding Patterns

**Figure 6.**
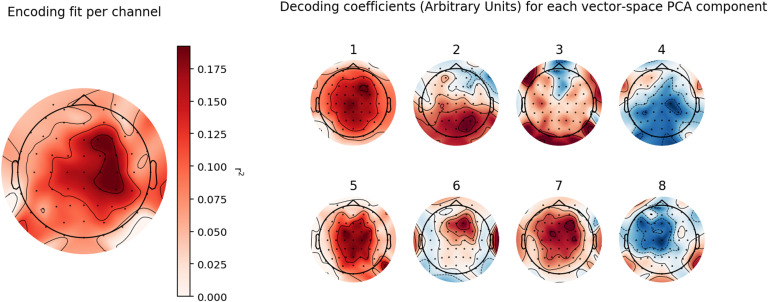
Visualization of the scalp distribution of encoding and decoding models. *Left*: Scalp distribution map showing the *r* ^2^ of the encoding model, for the N400 time window (300–500 ms), per electrode. *Right*: For each of the eight Kernel PCA components, the variance-normalized coefficients of the respective decoder (i.e., the forward model) are shown per electrode; that is, more red/blue coloring indicates that a more positive/negative going ERP leads to a higher prediction on the respective Kernel PCA score (German data set; compare Figure 5). These values are dimensionless.

By visualizing the spatial structure of these effects (see Figure 6), we find, very broadly speaking, that the spatial distribution of encoding performance as well as the informativity of electrodes for the decoding procedure partially, but not fully, overlap with the scalp distribution of the N400 effect known from other studies of semantic processing (Kutas & Federmeier, 2011). That is, we observe that encoding performance is highest at central electrodes near the midline, and that for at least four Kernel PCA components (1, 5, 7, and 8), the most important predictors in the decoding analysis are similarly found at central electrodes near the midline. However, other components are better predicted by other electrodes, perhaps indicating that different neural generators are involved in these representations.

## DISCUSSION

In this study, we have demonstrated that brain activity measured with EEG (more specifically, the distribution of ERP time courses over the scalp) encodes distributional vector representations of word meaning, as derived from nowadays well-established prediction-based distributional models. This result suggests that semantic vector spaces represent semantic relationships between words in a manner that is at least partly shared with how the brain represents word semantics. More specifically, words that occupy adjacent spaces in the high-dimensional vector space representations of these models also induce similar brain-activation states. These correspondences were largely the same for two experiments conducted in different languages (i.e., English and German) and requiring two different behavioral tasks. In addition, our results did not change substantially when we used a number of different implementations of distributional models.

On the one hand, our results indicate that distributional vector embeddings of word meaning allow accurate simulations of brain states (i.e., the “encoding” of word vectors in brain activity), indicating that word semantics, as expressed in word vectors, entrain brain states into predictable patterns. On the other hand, our data also demonstrate that vector-based word embeddings allow reading out (i.e., “decoding”) parts of the meaning of words from brain activity. This indicates that a semantic architecture at least partially similar to distributional word vector spaces is encoded in brain activity, even though these may not be directly interpretable semantic features. It is particularly noteworthy that the vector-space components that could be read out (decoded) from brain activity were independently described by trained linguists as corresponding to axes of meaning (rather than non-semantic aspects of word knowledge, such as a word’s length or frequency of occurrence). This is indeed expected if brain activity elicited during word processing contains representational information about word meaning and if the nature of this neural representation is at least partly related to the distributional nature of word vectors.

In the following, we discuss the implications of these results for the cognitive neuroscience of meaning, that is, for understanding the how, what, and when of representing the semantics of words in the human mind and brain. First, we discuss how the present results relate to various models of semantic representation. Then, we argue that some distinctions between these models (e.g., between feature- and exemplar-based architectures) are irrelevant, so that the core difference between models is the feature space in which they (perhaps implicitly) operate. In addition we review in somewhat more detail Jerry Fodor’s argument that *conceptual* feature spaces—unlike the not directly interpretable distributional word vector models we studied here—are implausible because they are circular (Fodor & LePore, 1999). From these considerations, combined with our empirical results, we elaborate that distributional models—like those whose fit to brain activity we tested here—are reasonable contenders for the architecture of the semantic system. Finally, we connect our results to previous findings and theories from the cognitive neurosciences, and argue that brain activity in the N400 time window reflects not only retrieval/integration-related “processing,” but also (context-independent) semantic “representations.”

### Models of Semantic Representations

What options for models of semantic representations are there? Distributional models like Word2vec have been proposed only recently, joining a long list of alternative proposals. “Empiricist state-space” theories (e.g., Churchland & Sejnowski, 1990) propose that word meaning can be understood as the ranking of a word on various experiential dimensions, that is, categories derived from our senses. An important role of not only the sensory, but also the motor system for the representation of semantics is suggested by “embodiment” theories (e.g., Gallese & Lakoff, 2005; Pulvermüller, 2013). These theories claim that we represent both concrete and abstract categories with reference to our sensory and motor systems. In addition, there are “nativist feature” theories, which propose that our mental concepts can be decomposed, although these features are not understood as experiential in nature, but innate (e.g., Pinker, 2008). Finally, “exemplar” *or* “prototype” theories (e.g., Edelman, 1995; Rosch, 1999) postulate that concepts are represented as, or with respect to, exemplars. Although prototypes must not necessarily correspond to objects or persons we have seen in the real world with our own eyes, they are constructed based on such experiential phenomena. Assigning a specific token to a semantic concept then corresponds to checking how similar it is to the respective prototype.

Under all these approaches, semantic representations can be conceptualized in matrix form, that is, geometrically as vectors or as points in high-dimensional state spaces (e.g., Churchland, 1993; Kriegeskorte & Kievit, 2013; Warglien & Gärdenfors, 2013). The disagreement between these models is about what the axes of the space or matrix correspond to: Nativist models assume they reflect innate, abstract protosemantic concepts (as those in Dowty, 1994; see also Pinker, 2008). Empiricist (Churchland, 1993; Prinz, 2004) as well as embodiment models (Pulvermüller, 2013) suggest experiential features, whereas in prototype/exemplar models (Edelman, 1995), exemplars take the role of the features—that is, a concept’s position is described with regard to its distance from other concepts.

Of interest, it has turned out to be irrelevant if these features are categorical or continuous (+ANIMATE vs. DEGREE OF ANIMACY; *cf.* Tissier, Gravier, & Habrard, 2018) in nature. Similarly, it turns out to be irrelevant if distances are represented with regard to other concepts (i.e., exemplars or prototypes) or to features. Conceptually, this can be seen by considering that a distance between two items corresponds to a consideration of feature overlap—in other words, the distance between two exemplars or prototypes implicitly refers to a feature space. If a different feature space is chosen, new distances result. Thus, exemplar models operate at least implicitly in a feature space, which has been formalized mathematically in the form of the so-called representer theorem (Schölkopf, Herbrich, & Smola, 2001; Jäkel, Schölkopf, & Wichmann, 2007; for a related development, see Nunez-Elizalde, Huth, & Gallant, 2019). This finding establishes that for a broad class of inference processes, the so-called primal and dual problems (in this case corresponding to feature- vs. exemplar-based encoding processes) are equivalent; conducting the inference in one space gives the same results as in the other (see also Figure 1, *bottom*). Thus, any query of the semantic model yields the same results for a feature-based representation as compared to a representation in an exemplar space defined by the same features. Moving from feature to exemplar space simply requires applying a “kernel function” (i.e., a similarity measure, yielding the distance between entities in feature space; this inspired our choice of Kernel in the decoding section). This implies for a quantitative evaluation of alternative models of semantic representations that the crucial question does not pertain to the general architecture of the model—that is, a featural versus exemplar-based architecture. Rather, it is critical to understand the type of features that define the semantic state space. We hoped to elucidate this by testing how well various feature spaces can predict the brain activity elicited during word perception.

We found that distributional word vector models allow the prediction (encoding) of human brain activity, and that this prediction is significantly better than prediction based on a classical taxonomic baseline model. This superiority of distributional vector-based representations to taxonomic models suggests that logical relations such as “x is a y” (hyponymy/hyperonymy) are not sufficient to grasp the neural implementation of semantic coding. Richer associations—perhaps including dimensions that resist verbal descriptions—are superior in explaining brain activity, and are in turn encoded in brain activity. In this sense, our work complements previous research on feature-based explanations of meaning representation (Fyshe, Sudre, Wehbe, Murphy, & Mitchell, 2012; Simmons, Hamann, Harenski, Hu, & Barsalou, 2008). In this sense, our work converges with the encoding of blood oxygen level–dependent responses to continuous, naturalistic language stimulation reported by Huth et al. (2016) but extends this work by demonstrating a systematic relationship between brain activation during isolated (i.e., context-free) word reading and distributional word vectors.

On the other hand, our “decoding” results suggest that the neural activity explained by distributional word vector models is at least in part genuinely semantic in nature. Some of the features we can decode are unsurprising; for example, concreteness is known to be a major determinant of lexical representation and was shown to modulate brain activity (Dufau et al., 2015). Others can be interpreted as evolutionarily relevant signals, that is, threat, food, or social aspects of meaning, which have been noted independently to affect word processing (Bentall & Kaney, 1989). However, the mere observation that a feature can be decoded from brain activity does not in itself mean that it is a major axis of the neural representation of meaning. The encoding process is bottlenecked on both sides by the quality of the vectorial representations (i.e., it is possible that a highly important dimension simply cannot be learned on data, or by models as those we use here), and by the SNR of the brain data. Our dimensionality reduction of the word vectors was based entirely on patterns of (co)variance within the word vectors themselves, not on what information is encoded in the neural signal. Put differently, the decoding procedure can only discover statistical relations between brain activity and semantic patterns identified independently of brain activity. Thus, although our results are suggestive of distributional semantics, they do not provide any finer insights into the specifics of human semantic representations.

Of interest, there are also indications that distributional, Word2vec class models can also yield inferior results compared to alternative models. For example, Huth et al. (2016) performed an analysis comparing their primary analysis to a Word2vec-based distributional model. Contrary to our results, these authors found that Word2vec explained fMRI activity above chance, but significantly worse than their primary model. The most obvious differences between these two studies are the recording modality (fMRI vs. EEG) and the stimulus coherence (narratives in Huth et al., 2016, as opposed to single words in our study). One important difference between EEG/ERP and fMRI is that fMRI recordings have superior SNR levels. This suggests that differences in SNR per se cannot explain why Word2vec performed better in our EEG/ERP study. A major difference is furthermore the superior temporal resolution of EEG. We thus speculate that the high temporal resolution of EEG/ERP may have allowed us to specifically identify the moment in time when processing of word meaning is performed. In contrast, fMRI conflates different cognitive processes into one data point, which may reduce its ability to capture the temporally circumscribed instantiation of meaning during word comprehension.

Another difference between the materials in our study and this previous work concerns the ongoing integration of words into phrasal and sentence contexts—contexts that were available to participants with the materials employed by Huth et al. (2016) but not with our single-word materials. Because many or even all words are polysemous (Hagoort et al., 2004), many of our stimulus words were ambiguous between multiple meanings, and some even between word classes (e.g., “fish,” which can function both as noun and verb). In contrast, word processing with constraining contexts is a different task, where only a narrow subset of the possible semantics associated with a word are evoked in order to possibly be integrated into the mental representation of the sentence. Moreover, continuous narratives consist of both content and function words, many of which carry their own semantics. This might lead to important differences in processing between continuous narratives and individual words. However, although this crucial difference may underlie differences between our findings and the results reported by Huth et al. (2016), the direction of the difference is surprising because intuitively one might have expected to find the context-dependent Word2vec analysis to perform superior for words presented in context. In the long run, theoretical work will have to develop a theory that can simultaneously accommodate both the integration of meaning in context, as well as the representation of meaning evoked by individual words presented in isolation.

We also note that we found vectors based on the original Word2vec implementation (also used by Huth et al., 2016) substantially inferior to the improved FastText-based vectors we employed. It is thus possible that improved distributional or taxonomic models will both yield new results. Finally, we would also like to point out that differences in task and material are highly likely to determine which feature spaces prove superior in different study contexts. For example, neural signatures of taxonomic judgment tasks are likely to favor taxonomic models. Future work will thus also have to explicitly explore the effect of such “design” variables on the encoding and decoding results.

Furthermore, it should be noted that the specific stimuli employed may have a strong impact on our results (as well as on other studies using similar model-based approaches): The extent to which results can be generalized beyond the given study is limited by the specific nature of the stimuli, which here consisted of short nouns and, for the German data, of clearly abstract or concrete words. For example, as an inherent limitation of distributional models, such models capture much less well the meaning of very low-frequency words. Because of the length restrictions of our experiment, we did not employ extremely long and rare words such as “heteroskedasticity,” for which a distributional model–based fit is expected to be worse. Similarly, little can be said about, for example, regular compounds.

### Circularity of Conceptual Features, and the Appeal of Distributional Models

In the literature on semantic architectures and representations, we have so far not found an example of a comprehensive list of semantic features—in the sense of a feature list that could clearly capture the semantics of any nontrivial set of word meanings. To the best of our knowledge, there is no such canonical list of semantic features with a claim to completeness. Put differently, theories of semantics (see preceding text) are proposals regarding the “kind” of features that define the representation of semantic meaning, but none of these models has so far spelled out testable proposals for these features. We argue that this hinders the empirical evaluation of the proposed theoretical models, as these models cannot generate quantitative and thus testable predictions without a clear definition of the involved features (i.e., without an explicit enumeration of the labels of the axes of the feature space).

Fodor and LePore (1999) and Fodor (1970) have argued that any enumeration of specific features would be futile, because any definition of concepts with features that are themselves conceptual in nature is either insufficient or trivial. For example, it would be unsatisfactory to define “cat” as having a feature +CAT. By this circular definition, the set of features would equal or exceed the number of word meanings. Instead, feature semantics strives to achieve a parsimonious description by identifying redundancies. Such an approach seems to work well for some showcase concepts such as “bachelor” (+MALE, +UNMARRIED). On a closer look, however, most words resist a decomposition into any convincing bundle of features as long as they do not become tautological. For example, “die” means BECOME DEAD, and “dead” means HAVE DIED, etc. (Fodor, 1970). Similarly, “cat” cannot be defined by any parsimonious set of features excluding the feature +CAT. Thus, a model of semantic representation where the discriminating features (also called axes or dimensions) are concept-like is—by virtue of circularity—implausible.

Already in 1997, Landauer and Dumais proposed that a practical approach (called latent semantic analysis, or LSA) for deriving vectorial representations of words is to enumerate in what contexts (e.g., documents and sentences) words occur, and then to identify latent variables underlying this massive *contexts* × *words* matrix. Although LSA is surprisingly effective, it is unlikely that this is similar to how humans actually learn; toddlers probably do not have the capacity to store verbatim thousands of paragraphs. Models like Word2vec make distributional vectors “learnable” by implicitly connecting them to predictive processing, that is, a process human brains are well known to be constantly engaged in (e.g., Clark, 2013). This offers a promising improvement on earlier models. The important aspect of Word2vec and LSA-style models, relative to classical conceptual feature space models, is that their axis labels are not conceptual, which allows them to evade the circularity critique. By this, we suggest, they become surprisingly plausible candidates for the representational architectures of the human mind. In addition, they have the benefit of being already available in implemented form—unlike competing models, which are often only specified conceptually. This, in turn, opens up a framework for robust and quantitative tests of theories of semantic representation. The data presented in the present study provide a natural extension of such conceptual considerations by empirically demonstrating the relationship between word vectors and brain states.

Proponents of grounded or embodied semantics (for a recent review, see Hauk & Tschentscher, 2013) would argue that the dimensions of representation of distributed models lack a biological grounding. However, these two approaches may in fact be surprisingly complementary. For example, Frome et al. (2013) have presented work indicating that combining perceptual and (text-based) distributional information may be a key aspect to rapid word acquisition (in their case demonstrating inference of semantics based on both word distributions and images). This suggests that combining statistical information about word context with the rich understanding of the physical world typical of human learners may be essential for improving the performance of distributional models to reach human-equivalent levels. For this reason, we prefer to see current empiricist distributional models (like investigated in the present work) not as an alternative to grounded theories of semantics. Instead, we think that they supplement one another, and integrating such perspectives could be a key step in furthering our understanding of human semantic knowledge.

### Meaning and the N400 Time Window

Our finding that semantic vector space information is encoded in brain activity measured between 300 and 500 ms after word onset has interesting implications for understanding the cognitive and neural processes underlying activity in the time window of the N400 component of the event-related brain potential. The N400 has in a large body of empirical research been shown to covary with the difficulty of semantic processing of words. In particular, it has been linked to lexical access and the integration of a word into a sentence context (Kutas & Federmeier, 2000, 2011). Here, we find that neurophysiological brain activity in the N400-time window contains information that is partially structured akin to distributional semantic models—in a situation without a context to integrate words into. This indicates that N400 time window activity reflects more than the difficulty of cognitive processes associated with retrieving word meaning and/or integrating it into the current context—which is the currently most widely accepted interpretation of this ERP effect (Kutas & Federmeier, 2011; Lau et al., 2008). Such a gradient of context-dependent processing difficulty is also mainly what previous attempts to encode word vectors in EEG activity have observed (Broderick et al., 2018; Ettinger et al., 2016). Instead, our findings indicate that EEG activity in the N400 time window also directly reflects a word’s meaning. This is not surprising; after all, one could even go so far as to say that the very purpose of words is to systematically shift the brain of the perceiver into certain states that represent their meaning. Thus, there “should” be a correspondence between the meaning of words perceived in isolation and the perceiver’s brain states. However, this was so far not directly accessible to typical empirical neurocognitive studies, as most previous research has focused on context-dependent “processes” rather than context-independent “representations.”

The observed encoding of semantic vector spaces in brain activity opens up a novel set of questions for researchers investigating the brain bases of meaning and language. Ideally, cognitive semantics and research on the neurobiology of language can jointly address the question of how words, after they have been processed (accessed, retrieved, integrated, and so on), are represented in brain activity. On the other hand, our results also suggest novel ways of investigating the corresponding cognitive architecture. For example, the encoding approach chosen here allows transcending factorial condition-contrast experiments, instead making it possible to directly compare representational frameworks with each other (Kriegeskorte & Kievit, 2013). Integrating our data with the currently available body of evidence indicates that processing-dependent aspects of semantics play out in approximately the same time window as representation-dependent aspects (i.e., roughly the N400 time window), suggesting that there may not be a strict boundary between representing and processing—as suggested by, for example, dynamicist models of semantic processing (Elman, 2009; Kutas & Federmeier, 2011; Lupyan & Lewis, 2017).

### Outlook

Distributional word vectors (learned from statistical patterns in texts) provide a demonstration of how a representational space sufficient to afford human semantic cognition could work. An important implication of our finding that semantic representations are encoded in brain activity (i.e., the event-related brain potential), and thus can also be decoded from EEG activity, is that “any” theory of semantics can be tested against neural data, to the extent that it can be quantified. That is, any independently motivated list of features could easily be encoded and the result compared to a suitable benchmark (such as the models tested here). Our results constitute one such benchmark; a research strategy for competing models should be to aim at producing higher portions of explained variance than reported here, that is, to beat the prediction accuracy obtained from distributional word vectors in the present work.

But is it plausible to assume that the brain computes a Word2vec-like analysis when learning words, or that it represents its semantic knowledge in abstract multidimensional vector form? Most certainly not. Existing methods, although powerful, are far from human-like in performance, even if they are trained on much larger corpora than children need for learning. And most models—including those tested here—are unimodal; that is, they do not derive any support from sensory experiences, generalization from other domains, or any cognitive priors, which would be a highly inefficient strategy for brains to use. We do not wish to suggest that mental semantics literally equal the Word2vec algorithm or similar distributional models. However, we agree with Landauer and Dumais (1997; see preceding text) that distributional word vector models avoid certain key problems of alternative approaches, most importantly the circularity critique developed by Fodor and LePore (1999). Combined with the successes of distributional models in decoding and encoding of brain activity, they establish a strong baseline for the modeling of word semantics–associated brain activity, which competing models must beat.

## ACKNOWLEDGMENTS

The authors wish to thank Jonathan Grainger and Stephane Dufau for making available the English EEG data set. Edvard Heikel organized the collection of the German EEG data. The MNE-Python Encoding team—in particular Chris Holdgraf, Eric Larson, Alexandre Gramfort, Denis Engemann, and Jean-Remi King—helped with the analysis code and conceptual discussion.

## FUNDING INFORMATION

C. J. Fiebach, FP7 Ideas: European Research Council (http://dx.doi.org/10.13039(100011199), Award ID: n° 617891.
